# DNA Hypomethylation of *MIR21* Drives Hsa-miR-21-5p Expression in High-Grade Meningiomas and Reshapes Transcriptomic Signatures of Oncogenic Pathways and Intercellular Communication

**DOI:** 10.3390/ijms27104403

**Published:** 2026-05-15

**Authors:** Paulina Kober, Szymon Baluszek, Beata Joanna Mossakowska, Izabella Myśliwy, Biniyam Tsegaye, Artur Oziębło, Tomasz Mandat, Mateusz Bujko

**Affiliations:** 1 Department of Experimental Oncology, Maria Sklodowska-Curie National Research Institute of Oncology, 02-781 Warsaw, Poland; paulina.kober@nio.gov.pl (P.K.); szymon.baluszek@nio.gov.pl (S.B.); beata.mossakowska@nio.gov.pl (B.J.M.);; 2 Department of Neurosurgery, Maria Sklodowska-Curie National Research Institute of Oncology, 02-781 Warsaw, Poland; artur.ozieblo@nio.gov.pl (A.O.); tomasz.mandat@nio.gov.pl (T.M.)

**Keywords:** DNA methylation, hsa-miR-21-5p, meningioma, tumor microenvironment, border-associated macrophages

## Abstract

Meningiomas are the most common intracranial tumors. DNA methylation analysis in benign and aggressive meningiomas showed decreased *MIR21* methylation and overexpression of hsa-miR-21-5p in atypical and anaplastic tumors. Transcriptomic analysis of distinct WHO grade meningiomas showed multiple predicted hsa-miR-21-5p target genes as differentially expressed. They were mainly related to processes of intercellular and intracellular signaling. Intercellular communication in meningioma was investigated using the deposited scRNA-seq dataset and deconvolution of our RNA-seq data. We found WHO grade-related differences in the microenvironment including inverse correlation between the count of border-associated macrophages (BAM) and the level of hsa-miR-21-5p. Single-cell transcriptomics suggests the role of interleukin 6 in direct communication between tumor cells and BAMs. *IL6R* and *IL6ST* are predicted targets of hsa-miR-21-5p downregulated in atypical/anaplastic meningiomas. IL6R downregulation was also confirmed by immunohistochemistry. Hsa-miR-21-5p enhanced proliferation and viability of KT21-MG1 meningioma cells and showed a regulatory effect on *IL6R*, *IL6ST* and other predicted target genes *TIMP3*, *PIK3R*, *RHOB*, and *SASH1* by interacting with 3′UTRs. DNA hypomethylation-related overexpression of hsa-miR-21-5p contributes to aggressive meningioma growth by interaction with multiple target genes, and probably affects microenvironment communication between meningioma cells and BAMs by lowering the IL6R level in tumor tissue.

## 1. Introduction

Meningiomas are one of the most common intracranial tumors in adults [1]. They are diagnosed more frequently in people over the age of 65 and much more frequently in women. The histopathological spectrum of meningiomas is diverse, and the vast majority of meningiomas (80%) meet the histological criteria for slow-growing benign malignancies, corresponding to WHO Grade (G) I. WHO GII tumors (mainly atypical meningiomas) and GIII tumors (mainly anaplastic meningiomas) account for about 18.3% and 1.6% of all meningiomas, respectively [1]. Patients with the most frequent benign meningiomas generally have good prognosis [1]. Surgical treatment is effective in most cases; however, benign meningiomas tend to progress to more aggressive subtypes. WHO GII and GIII tumors have an invasive phenotype with increased risk of recurrence, invasion of normal meninges, skull or brain structures, and a shorter survival time [2].

In recent years, significant advances have been made in understanding the molecular basis of meningiomas, including the role of epigenetics [3]. The results of genome-wide DNA methylation patterns contributed to the development of the molecular subclassification of meningiomas and have shown the role of the aberrant methylation pattern in the expression of tumor-related genes [4]. In our research, we also found aberrant methylation in genes encoding miRNAs, which can also play an important role in the pathogenesis of these tumors [5].

MiRNAs are class of small, endogenous, non-coding RNA molecules, which are involved in the negative regulation of gene expression at the post-transcriptional level [6]. Due to their important regulatory role, alterations in miRNA expression contribute to neoplastic transformation [6]. By analogy with the classification of cancer-related proteins, some miRNAs are considered tumor suppressors, and their downregulation in tumors may lead to the overexpression of target protein-coding genes of oncogene nature. In contrast, the upregulation of oncogenic miRNAs in tumors results in the downregulation of tumor suppressor genes [7]. Many such miRNAs were found to be aberrantly expressed in human meningiomas [8]. Hsa-miR-21 is among the known oncogenic miRNAs with multiple roles in carcinogenesis identified so far [9]. Epigenetic deregulation of the *MIR21* gene was also reported in a few types of cancer [10].

## 2. Results

### 2.1. MIR21 Promoter DNA Hypomethylation and the Expression of Mature Hsa-miR-21 in Meningiomas

**Figure 1:**
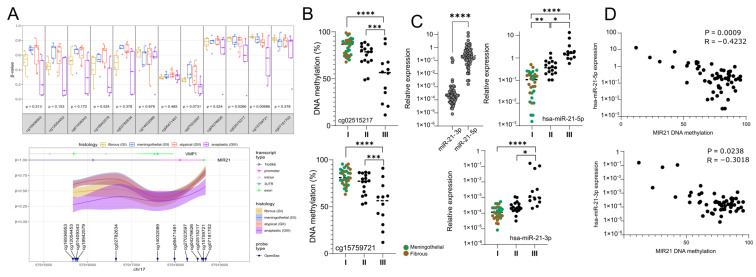
DNA methylation at *MIR21* as well as the expression of hsa-miR-21-3p and hsa-miR-21-5p in WHO GI (meningothelial and fibrous), GII (atypical) and GIII (anaplastic) meningiomas. (**A**) Analysis of data from previous methylation profiling in meningioma samples with HM450K (GSE241956); (**B**) bisulfite pyrosequencing-based evaluation of particular CpGs at the *MIR21* locus; (**C**) the expression levels of hsa-miR-21-5p and hsa-miR-21-3p in meningiomas of distinct WHO grade; (**D**) the correlation between hsa-miR-21 expression and *MIR21* methylation levels. Asterisks indicate the statistical significance of differences (* *p* < 0.05, ** *p* < 0.01, *** *p* < 0.001, **** *p* < 0.0001).

We used data from our previous DNA methylation profiling with Human450K arrays in meningioma samples (10 benign, 8 atypical and 6 anaplastic meningiomas) to compare the methylation level at the *MIR21* locus in WHO GI, GII and GIII meningiomas. We observed that two CpGs located in the core promoter (cg02515217) and the transcribed sequence (cg15759721) of the *MIR21* gene were significantly hypomethylated in higher grade meningiomas (*p* = 0.0266 and *p* = 0.00686, respectively) (Figure 1A). DNA methylation at these two CpGs was measured in a series of 62 meningiomas with bisulfite pyrosequencing assay. Comparison of tumors of distinct histological diagnosis confirmed that the methylation level of *MIR21* is gradually decreased in GII atypical and GIII anaplastic tumors compared to benign meningiomas (Figure 1B). The same tumor samples were subjected to measurement of the expression levels of hsa-miR-21-3p and hsa-miR-21-5p. We observed a notably higher expression of hsa-miR-21-5p than hsa-miR-21-3p in tumor samples and both mature hsa-miR-21 particles had higher expression in WHO GII atypical and WHO GIII anaplastic meningiomas than in GI tumors (Figure 1C). The expression of both hsa-miR-21-5p and hsa-miR-21-3p was inversely correlated with *MIR21* methylation levels; however, the correlation coefficient was notably higher for the -5p particle (Figure 1D).

### 2.2. Relationship Between the Increased Hsa-miR-21-5p Level and Gene Expression in Meningiomas

**Figure 2:**
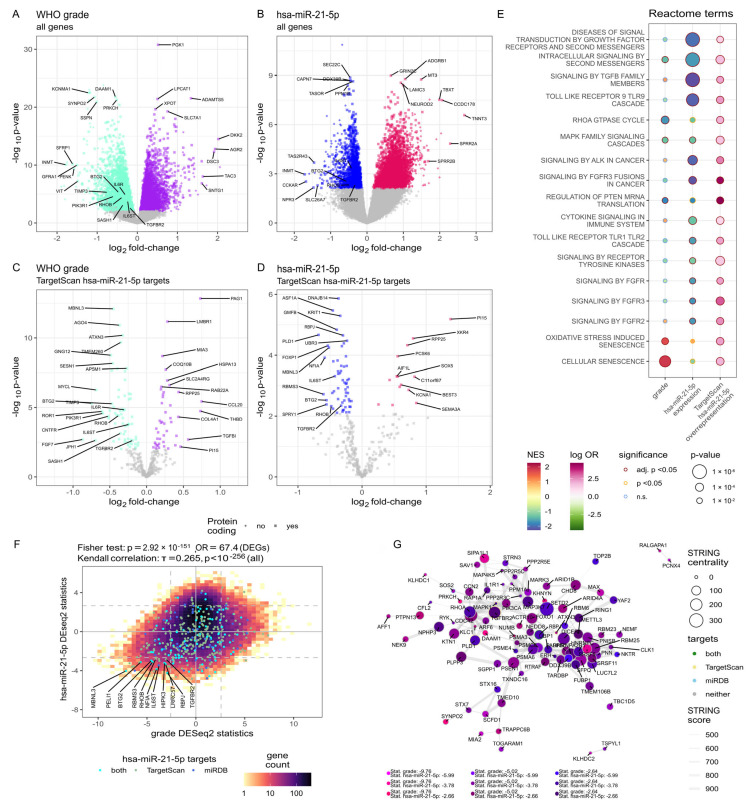
Results of RNA sequencing in meningioma samples and investigation of the overlap between hsa-miR-21-5p targets and genes exhibiting WHO grade-associated expression. Differential expression, volcano plots of genes with regard to: (**A**) meningioma grade; (**B**) hsa-miR-21-5p expression; (**C**) grade, subset of TargetScan hsa-miR-21-5p targets; and (**D**) hsa-miR-21-5p expression, subset of TargetScan hsa-miR-21-5p targets. (**E**) Reactome gene set enrichment analysis results. (**F**) Intersection of DEGs and hsa-miR-21-p5 (**G**) protein–protein interaction network of DEGs associated with lower grade and lower hsa-miR-21-5p expression, based on the StringDB network. ns—not significant.

Observed differences in *MIR21* methylation and expression among tumors of distinct grades prompted us to investigate the overlap between hsa-miR-21-5p targets and genes exhibiting WHO grade-associated expression. Sixty meningioma samples included in this study met RNA quality criteria and were subjected to RNA sequencing (RNA-seq); 56 were retained for analysis following data quality control, as described previously [11]. Differential expression analysis, with WHO grade as numeric values, revealed 5518 protein-coding genes with grade-related expression levels, as already reported [11] and summarized in Figure 2A. Among these differentially expressed genes (DEGs), 134 were predicted hsa-miR-21-5p targets based on TargetScan 2.0 and miRDB (Figure 2C; Supplementary Table S2).

According to TargetScan 2.0 predictions, the human transcriptome contains 384 hsa-miR-21-5p-targeted mRNA transcripts, whereas miRDB indicates its 154 target mRNAs (92 common for these two sources). Following assessment of hsa-miR-21-5p levels in each tumor sample, we found that a total of 3423 genes exhibited expression levels correlated with this miRNA (Figure 2B), including 85 predicted hsa-miR-21-5p targets (Figure 2D), as detailed in Supplementary Table S2.

We applied gene set enrichment analysis (GSEA) using the Reactome database to functionally characterize genes with hsa-miR-21-5p-correlated expression as well as the genes with WHO grade-related expression. This approach enabled the identification of processes associated with hsa-miR-21 and tumor WHO grade, which were subsequently filtered for those significantly overrepresented with putative hsa-miR-21-5p targets. As a result, predicted hsa-miR-21-5p targets in meningiomas were found to be enriched in processes related to intercellular communication and signaling by cytokines (e.g., IL6, TGFB, NTRKs and FGF), intracellular signaling through second messengers and PI3K and MAPK pathways (Figure 2E; Supplementary Table S3). Consistently, putative hsa-miR-21-5p target genes that were either differentially expressed across meningiomas grades or significantly correlated with miRNA expression were also enriched in these classes of processes (Figure 2F, details in Supplementary Table S3). Key genes central within these processes were identified using a stringDB protein–protein interaction (PPI) network (Figure 2G). Among TargetScan hsa-miR-21-5p targets, *RBPJ* and *TGFBR2* emerged as central nodes in this network.

### 2.3. Hsa-miR-21-5p and Tumor Microenvironment and Cellular Communication

**Figure 3:**
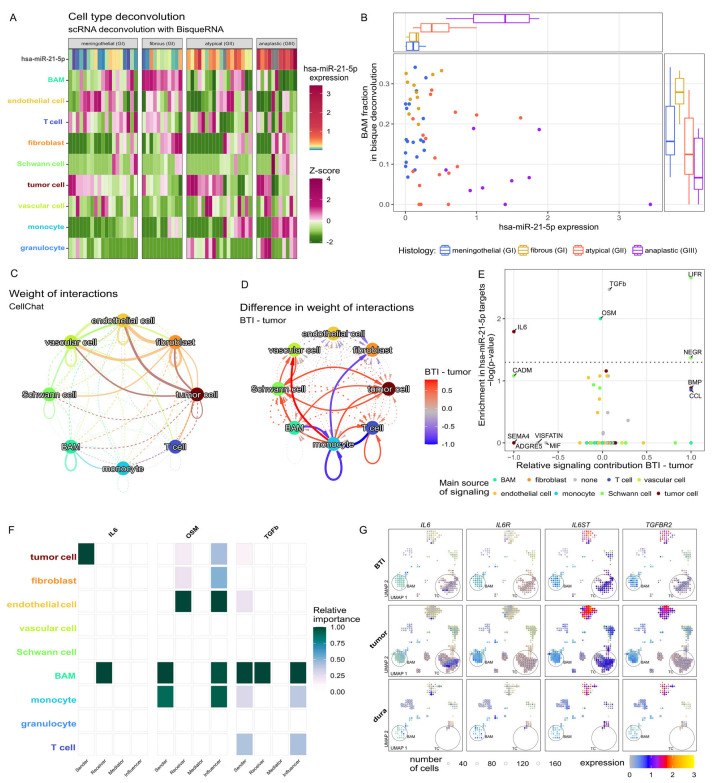
Determining the role of hsa-miR-21-5p in intercellular communication in meningioma based on the analysis of RNA-seq and scRNA-seq (publicly available GSE183655 dataset). (**A**) Deconvolution of bulk RNA-sequencing samples to cell types by Bisque. Note that border-associated macrophages (BAMs) are significantly associated with both grade and hsa-miR-21-5p concentration in the sample. (**B**) Correlation of BAMs with hsa-miR-21-5p level. Cell–cell communication network by CellChat in the scRNA-seq samples. (**C**) Weight of interactions in tumor samples. (**D**) Difference in interaction weight between tumor and brain-tumor interface (BTI) samples. (**E**) CellChat term enrichment in hsa-miR-21-5p targets. (**F**) Information flow in the selected (tumor or BTI-specific) pathways; note that specifically BAMs (and not monocytes) are dominant senders of TGFb signaling and exclusive receivers of IL6 and TGFb signaling, while both BAMs and monocytes contribute to the OSM pathway. (**G**) Gene expression of *IL6*, *IL6R*, *IL6ST*, and *TGFBR2* in cells of a given type. BAM—border-associated macrophages; TC—tumor cells (both indicated by circles). The cell clusters are described in detail in the Supplementary Figure S1 and previous article [11].

Since we identified processes of intercellular communication linked to hsa-miR-21-5p overexpression, we investigated the potential role of this miRNA in the regulation of the meningioma microenvironment. We reanalyzed publicly available data from single-cell RNA sequencing from benign meningioma samples (GSE183655) to characterize the cellular composition of this tumor, identifying 22 distinct cellular clusters of tumor cells, fibroblasts, endothelial cells, vascular cells, Schwann cells, border-associated macrophages (BAMs), monocytes, granulocytes, T-cells, B-cells, microglial and glial cells as previously described [11]. Transcriptomic profiles of these cell types were used for deconvolution of our RNA-seq data from meningioma samples of various WHO grades [11]. Since hsa-miR-21-5p levels were quantified in each of these tumor samples, we examined the correlations between the estimated abundance of each microenvironment component and hsa-miR-21-5p expression level (Figure 3A). The estimated count of BAMs showed significant inverse correlation with hsa-miR-21-5p expression (Figure 3B).

We modeled a cell–cell communication network using GSE183655 scRNA-seq data and CellChat. The weight of interactions in tumor samples, as well as differences in interaction weight between tumor and brain-tumor interface (BTI) samples, were calculated. Overall interactions weights are depicted in Figure 3C. Observed changes in the communication indicated increased signaling to tumor and vascular cells in the BTI, alongside decreased monocyte signaling (Figure 3D). To enhance hsa-miR-21-5p specificity, genes in CellChat terms were enriched for predicted hsa-miR-21-5p targets from TargetScan (Figure 3E). The results indicated direct, specific communication between meningioma cells (signal senders) and BAMs (signal receiver) via interleukin 6, with *IL6* gene expressed by tumor cells and *IL6R* by BAMs (Figure 3F). According to the scRNA-seq data, expression of both genes was notably higher in meningioma cells and BAMs in tumor compared to tumor-interface and normal dura, suggesting this signaling as part of the intratumoral communication network. Expression of the predicted hsa-miR-21-5p targets involved in this communication (*IL6*, *IL6R*, *IL6ST*, and *TGFBR2*) across cell types is presented in Figure 3G.

**Figure 4:**
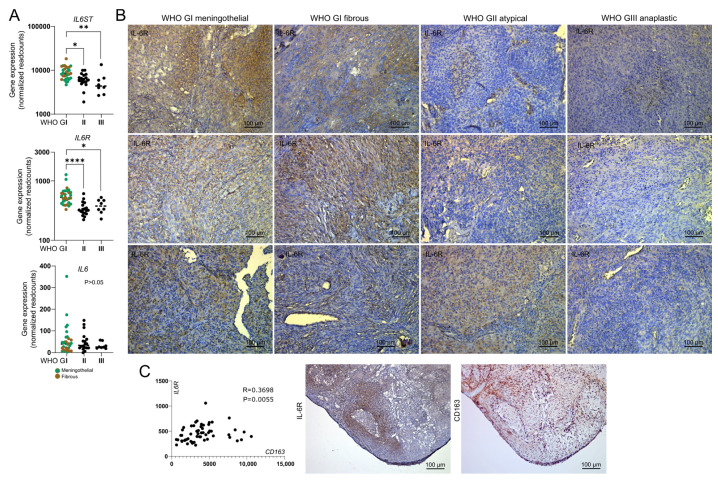
The expression of interleukin 6 receptor in meningiomas. (**A**) Comparison of the expression of *IL6ST*, *IL6R* and *IL6* genes in meningiomas of various WHO grades. (**B**) Representative results of immunohistochemical staining for IL6R in meningiomas (magnification ×200). (**C**) Correlation between *IL6R* and *CD163* genes in meningiomas and the protein expression of IL6R and CD163 (macrophage marker) in a benign meningioma sample. Asterisks indicate the statistical significance (* *p* < 0.05, ** *p* < 0.01, **** *p* < 0.0001).

Predicted hsa-miR-21-5p target genes are enriched for genes related to Reactome interleukin-6 signaling (Supplementary Table S3). Importantly, posttranscriptional regulation of *IL6R* by hsa-miR-21-5p has already been validated in vitro [12,13,14]. We observed downregulation of *IL6R* and *IL6ST* (IL6 receptor-encoding genes) in meningiomas of higher WHO grade compared to benign ones, with no difference in *IL6* expression (Figure 4A). Immunohistochemical staining for the IL6R protein confirmed its downregulation in high-grade meningiomas, with the highest expression in WHO grade I tumors and the lowest in anaplastic (grade III) meningiomas (very low in 4/5 grade III tumors). Representative staining results are presented in Figure 4B.

Our RNA-seq data revealed some co-expression between *IL6R* and *CD163* (macrophage marker) genes, with moderate correlation in their expression levels (Spearman R = 0.37; *p* = 0.0055) (Figure 4C). Moreover, immunoreactivity for IL6R and CD163 in the same tumor sample showed spatial co-expression of these proteins, consistent with the observations from single-cell transcriptomics indicating *IL6R* expressed predominantly in BAMs. Altogether, these findings suggest reduced communication between meningioma cells and BAMs communication in higher grade tumors, coinciding with elevated hsa-miR-21-5p expression, which is a negative regulator of *IL6R* and *IL6ST* post-transcriptionally.

### 2.4. The Effect of Upregulation of Hsa-miR-21-5p Level In Vitro

**Figure 5:**
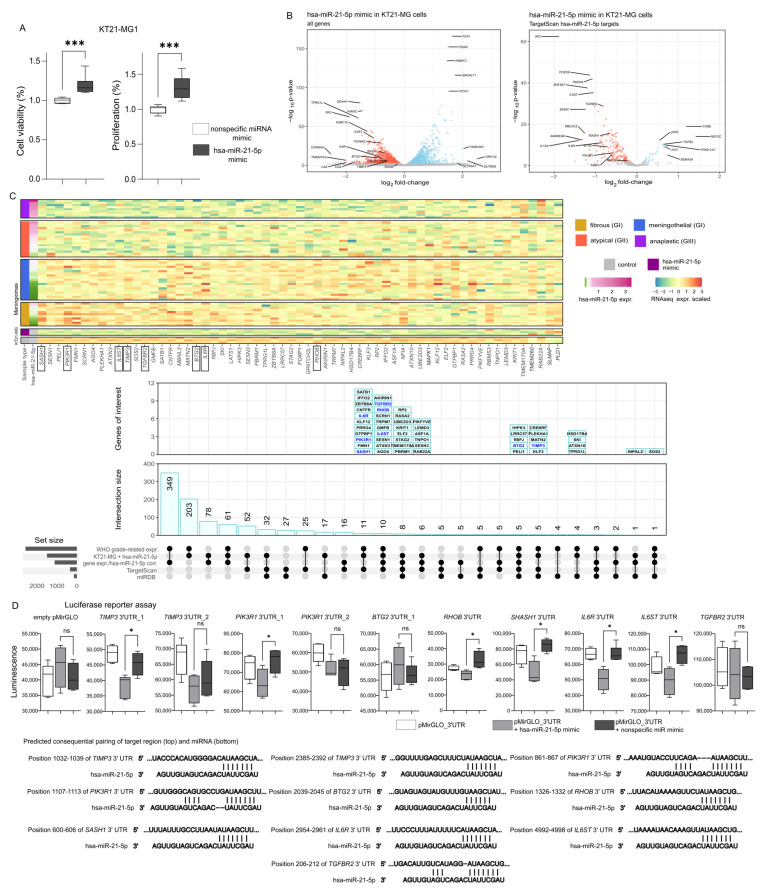
Results of in vitro examination of the role of hsa-miR-21-5p. (**A**) Effect of hsa-miR-21-5p mimic on the cell viability and proliferation of human meningioma cell lines KT21-MG1. (**B**) Difference in gene expression in KT21-MG1 cells treated with hsa-miR-21-5p mimic and negative control mimic. (**C**) The expression level of genes of interest in KT21-MG1 cells treated with hsa-miR-21-5p mimic and control cells and in meningioma samples, and summary of the genes identified as related to meningioma grade, co-expressed with hsa-miR-21-5p, determined as hsa-miR-21-5p targets and differentially expressed in hsa-miR-21-5p mimic treated and control cells. Target genes selected for luciferase reporter gene assay validation are marked in boxes below heat map and in blue. (**D**) Results of luciferase reporter assays verifying the interaction between the fragment of 3′UTRs of genes of interest and hsa-miR-21-5p mimic. Asterisks indicate the statistical significance of differences (* *p* < 0.05, *** *p* < 0.001, ns—not significant).

The role of hsa-miR-21-5p was also examined in a meningioma cell line to determine its effects on cell phenotype and interactions with selected target genes. KT21-MG1 meningioma cells were transfected with hsa-miR-21-5p miRNA mimic to explore its influence on cell viability (MTT test) and proliferation (BrdU incorporation-based test). We observed increased proliferation and viability in KT21-MG1 cells treated with hsa-miR-21-5p mimic compared to cells treated with a negative control mimic (Figure 5A), confirming the oncogenic role of this miRNA.

Then we explored the effect of hsa-miR-21-5p upregulation on gene expression in KT21-MG1 cells. The cells were treated with hsa-miR-21-5p or control mimic and the gene expression profiles across experimental conditions were analyzed using RNA-seq. Transfection with hsa-miR-21-5p altered the expression of 159 genes (including 133 downregulated and 26 upregulated genes) which are the targets of this miRNA, according to TargetScan and/or miRDB predictions (Figure 5B).

By further integrating the transcriptomic results of the in vitro experiment and the analysis of meningiomas, we identified 61 predicted hsa-miR-21-5p targets meeting at least two of three criteria: differential expression in the KT21-MG cell experiment; WHO grade–related expression; and expression correlated with hsa-miR-21-5p levels in tumors. These results are visualized in Figure 5C and detailed in Supplementary Table S4. Based on a literature review, we selected eight of these genes whose functions may contribute to meningioma pathogenesis. Next, the direct interaction between hsa-miR-21-5p and the mRNA of selected genes, namely *TIMP3*, *PIK3R*, *BTG2*, *RHOB*, *SASH1*, *IL6R*, *IL6ST* and *TGFBR2*, were tested in vitro.

We determined the target sites in 3′UTR of each gene using TargetScan 8.0, and then tested the interaction between the putative binding motif and hsa-miR-21-5p using luciferase reporter assay in KT21-MG1 cells. We confirmed that hsa-miR-21-5p interacts with one of two putative binding motifs in *TIMP3* and *PIK3R* as well as with the target sites in *RHOB*, *SASH1*, *IL6R* and *IL6ST*; however, the experiment did not confirm the interaction with *BTG2* and *TGFBR2* (Figure 5D).

## 3. Discussion

The expression of the genes encoding miRNAs can be regulated by epigenetic mechanisms [15,16], and abnormalities in this regulation can alter the expression of mature miRNAs with cancer-related properties [17]. Numerous miRNAs epigenetically dysregulated via aberrant DNA methylation have been identified in human cancers [17]. MiR-21 is a well-recognized oncogenic miRNA [9] and DNA hypomethylation at the *MIR21* locus was found in breast, lung, bladder, pancreatic, renal and kidney cancers [10], as well as thyroid cancer [18] and pituitary tumors [19]. These studies consistently link overexpression of hsa-miR-21 to *MIR21* hypomethylation in tumors. Accordingly, our results showed a progressive decrease in DNA methylation and an increase in the expression of *MIR21* in atypical and anaplastic meningiomas compared to benign ones, suggesting a role of this miRNA in the aggressive growth and progression of meningiomas. Two prior studies evaluated the expression of hsa-miR-21 in meningiomas [20,21]. Its significant upregulation in WHO grade II meningiomas was found in one of them [20]. We noticed that hsa-miR-21-5p is the most abundant mature miRNA from the *MIR21* locus with the most explicit grade-related overexpression (as assessed with both fold change and significance criteria); hence, we focused our research primarily on hsa-miR-21-5p.

According to target prediction methods, hsa-miR-21-5p interacts with multiple protein-encoding transcripts, and indeed many functionally diverse targets have been validated in previous research [9,22]. This miRNA alters the biology of many human cancers affecting a variety of pathways and molecular mechanisms in a tumor-type-specific manner [9,22]. To explore its role in meningiomas, we analyzed tumor transcriptomes and identified multiple putative hsa-miR-21-5p target genes that were differentially expressed across WHO grades and/or correlated with hsa-miR-21-5p levels. These genes were primarily associated with the processes of intracellular signaling (of MAPK, FGFR, BMP, SMAD and ALK pathways as well as toll-like receptor cascade) and intercellular communication (cytokine and TGFβ signaling)—which are pathways that have been already found to be regulated by hsa-miR-21 in numerous cancers [9,22,23,24]. We acknowledge that in meningiomas, hsa-miR-21-5p exerts a multifaceted role as it does in many other human neoplasms [9,22,25].

Given the complex cellular composition of tumor tissue and the role of the tumor microenvironment in meningiomas [26], we wanted to take a closer look at the probable role of hsa-miR-21-5p in the context of tumor heterogeneity. Deposited, freely available data from scRNA-seq of benign meningiomas were used to characterize tumor microenvironment composition and to assess the abundance of specific cellular populations in our meningioma samples with a RNA-seq deconvolution method. Interestingly, we found an inverse correlation between the hsa-miR-21-5p expression level and content of BAMs, which were found as the most profuse macrophage subtype in meningioma. BAMs, along with microglia, are a class of natural brain tissue-resident macrophages [27]. While microglia reside basically in brain parenchyma, BAMs are mostly located at the blood–brain and blood–cerebrospinal fluid barriers and in the meninges [28]. The role of BAMs in meningioma is unknown, and our deconvolution data are the first to indicate their depletion in higher grade tumors versus benign tumors.

The role of hsa-miR-21 in the regulation of macrophages was observed in many biological contexts, including neoplasia. Tumor-cell-derived exosomes preloaded with mature hsa-miR-21-5p regulate the macrophages of the tumor microenvironment [29,30,31,32,33,34]. Exogenous hsa-miR-21-5p can affect various pathways in tumor macrophages by targeting genes such as *RHOB* [31], *PELI1* [34], *SP-1* [33], *IRF-1* [30] or *PTEN* [29]. Silencing or *TGFBR2* in macrophages by exogenous hsa-miR-21-5p was also reported [35]. Conversely, macrophage-derived exosomal hsa-miR-21-5p may affect cancer cells, as observed in renal and pancreatic cancers [36,37].

Cell–cell communication modeling in meningioma indicates that interleukin 6 signaling may be specifically involved in direct communication between meningioma tumor cells and BAMs. According to scRNA-seq data, meningioma cells express *IL6*, while BAMs are the cells that express the highest *ILST* and *IL6R* level among all the cell populations identified in tumor tissue. The expression of interleukin 6 in meningioma tumor cells has also been previously observed by immunohistochemical staining of tumor tissue [38] and in cultured meningioma cells [39], aligning with our observations. Our immunostaining of a meningioma sample with antibody for IL6R shows the highest immunoreactivity at the areas of the highest expression of CD163 (a macrophage marker), supporting BAM-specific expression of *IL6R*—though we note that CD163 is not BAM-exclusive. The role of interleukin 6 signaling in meningioma-associated macrophages was also noted in another scRNA-seq study [40].

Both *IL6R* and *IL6ST* genes that encode proteins of the interleukin 6 receptor are the targets of hsa-miR-21-5p, as confirmed in our study on KT21-MG1 cells and prior studies on 293T epithelial cells [12], mouse primary myoblasts [13] and endothelial progenitor cells exposed to breast-cancer-cell-derived hsa-miR-21 [14]. Therefore, our results in the light of literature data suggest a mechanism where meningioma tumor cells regulate natural BAMs through interleukin 6 signaling. Overexpression of hsa-miR-21-5p in atypical and anaplastic meningiomas likely disrupt the communication between tumor cells and BAMs by targeting its *IL6R* and *IL6ST* transcripts in macrophages. We speculate that these macrophages restrain meningioma aggressiveness and the loss of IL6-related communication, through reduced IL6R expression in BAMs (without changing the interleukin 6 level in tumor tissue), contributing to their indolence and lowering their abundance. We cautiously propose this model, as it is primarily grounded on transcriptomic data in the context of the literature. Functional experiments on the interaction between meningioma cells and macrophages were beyond the scope of our study, and the lack of biological validation should be considered a notable limitation.

Our in vitro results in KT21-MG1 cells reinforce the multifaceted role of hsa-miR-21-5p. We found that hsa-miR-21-5p decreases the expression of some target genes that are also downregulated in higher grade meningiomas, including *TIMP3*, *PIK3R1*, *BTG2*, *RHOB*, *SASH1*, *IL6R*, *IL6ST* and *TGFBR2*—the genes with a role in human cancers [41,42,43,44,45] including meningiomas [46,47]. These miRNAs interact with 3′UTR of most of these genes. Using KT21-MG1 cells, we did not succeed to confirm binding of hsa-miR-21-5p to specified 3′UTR fragments of *BTG2* and *TGFBR2*, suggesting the indirect effect on these two genes; however, such an interaction was verified by others [23,48]. All these mentioned genes are expressed at various levels in distinct cellular components of meningioma according to scRNA-seq data, so we assume that hsa-miR-21-5p can influence both tumor cells and various elements of the microenvironment. The important question we were unable to answer is which of the cellular tumor components produces this miRNA. Most of the studies on the role of hsa-miR-21 in cancer assume its overexpression in cancer cells [10]. There are, however, results indicating that cancer cells can be targeted by hsa-miR-21-5p excreted by tumor-associated macrophages as well [49]. We propose that in meningiomas, epigenetically dysregulated hsa-miR-21-5p exerts diverse, pleiotropic effects consistent with observations in other cancer types [9]. We acknowledge that a complete characterization of the role of this miRNA would require further detailed functional studies addressing the relevance of its particular target genes. The involvement of hsa-miR-21 in cell–cell communication, demonstrated in previous studies and partially supported by our transcriptomic analysis, should be particularly considered in future research.

## 4. Materials and Methods

### 4.1. Patients and Tissue Samples

Sixty-two archival formalin-fixed paraffin-embedded (FFPE) meningioma samples from patients who underwent surgical tumor resection at the Maria Sklodowska-Curie National Research Institute of Oncology in Warsaw were used. The study group included 32 WHO GI, 18 GII and 12 GIII meningiomas. All tissue samples were histologically examined for a review of diagnosis and selecting a representative tissue section suitable for molecular analysis. Patients’ characteristics are listed in Table 1. RNA and DNA were isolated from FFPE tissue using a RecoverAll™ Total Nucleic Acid Isolation Kit for FFPE [Thermo Fisher Scientific, Waltham, MA, USA], measured using NanoDrop 2000 [Thermo Fisher Scientific, Waltham, MA, USA] and stored at −70 °C.

### 4.2. Ethics

The study was conducted in accordance with the Declaration of Helsinki and was approved by the Ethics Committee of the Maria Sklodowska-Curie National Research Institute of Oncology, Warsaw, Poland (approval no. 13/2019). Informed consent was obtained from all study participants.

### 4.3. DNA Methylation Analysis

Previously generated data on genome-wide methylation in meningiomas (10 benign (WHO GI), 8 atypical (WHO GII) and 6 anaplastic tumors (WHO GIII)) obtained with HumanMethylation 450 K Infinium Methylation BeadChip (Illumina, San Diego, CA, USA) was used (Gene Expression Omnibus; GSE241956) [50]. Data were analyzed using the minfi Bioconductor package [51].

The DNA methylation level of the *MIR21* locus covering cg02515217 and cg15759721 CpG sites was determined using bisulfite pyrosequencing, as previously [5]. One microgram of DNA was bisulfite-treated using an EpiTect kit (Qiagen, Hilden, Germany). A PCR reaction was performed in a volume of 30 μL containing 1×PCR buffer, 2 mM MgCl_2_, 0.25 mM dNTPs, 0.2 μM of each primer and 0.5 U of FastStart DNA Polymerase (Roche Applied Science, Mannheim, Germany). The following cycling conditions were used: 94 °C for 3 min, followed by 40 cycles of 30 s at 94 °C, 30 s at 52 °C and 30 s at 72 °C with a final elongation of 7 min at 72 °C. PCR products were purified using PyroMark Q24 Vacuum Workstation (Qiagen) and analyzed using PyroMark Q24 System (Qiagen), according to the manufacturer’s protocol. The sequences of the oligonucleotides are listed in Supplementary Table S1.

### 4.4. Measurement of miRNA Relative Expression Levels

Relative expression levels of mature miRNA were determined with a miScript miRNA PCR System (Qiagen). Total RNA was reverse-transcribed using a miScript II RT Kit (Qiagen), while a miScript SYBR^®^ Green PCR Kit (Qiagen) with miScript primer assays was used for PCR amplification, as previously [5]. The following miScript primer assays were used: MS00009079 (for hsa-miR-21-5p) and MS00009086 (for hsa-miR-21-3p), as well as MS00033740 (for snRNA RNU6B) that served as reference.

### 4.5. RNA Sequencing and Data Analysis

RNA isolated from 62 FFPE meningioma samples was subjected to RNA sequencing (RNA-seq) as already described [11]. Sequencing reads were aligned to the hg38.p11 genome using STAR. Then, quality control was performed with RseQC v5.0.3, and transcript integrity numbers (TINs) were determined. Samples with less than 15% of reads aligned to the genome were discarded, and reads were normalized with DESeq2 v1.44; TIN, the fraction of reads aligned to the genome, and median reads per gene were treated as batch effects. DEGs were obtained for tumor grade and hsa-miR-21-5p level, assessed in the samples; tumor grade and hsa-miR-21-5p expression were treated as numeric values for the DESeq2 algorithm to follow. Genes with an adjusted *p*-value < 0.05 were considered significantly differentially expressed. Predicted hsa-miR-21-5p targets were downloaded from the TargetScan 8.0 [52] database and miRDB database [53]. DEGs were enriched in terms from MsigDB v2024.1 [54] by fgsea v1.30.0 [55]. Targets for hsa-miR-21-5p were also enriched in these terms utilizing overrepresentation analysis (fora function in fgsea v1.30.0) [55]. Next, correlation was calculated between grade and hsa-miR-21-5p differential expression statistics. Finally, the String DB PPI database was utilized to search for the most important genes from a functional point of view; tidygraph v1.3.1 was utilized for visualization and estimating most central nodes in the PPI network.

For the in vitro experiments, total RNA from cultured cells was isolated using the High Pure RNA Isolation Kit (Roche Applied Science). For each sample, 1 µg RNA was used for poly(A)-enriched library preparation (Novogene, Beijing, China). Library quality was assessed with the Agilent Bioanalyzer 2100 (Agilent Technologies, Santa Clara, CA, USA). Sequencing was performed on an Illumina NovaSeq 6000 (150-bp paired-end; ≥30 million read pairs per sample) by Eurofins Genomics (Ebersberg, Germany). Reads were aligned to the hg38.p11 genome using STAR 2.7.11b. Genes significantly differentially expressed (adjusted *p* < 0.05) between the cells treated with miRNA mimic and control cells were obtained with DESeq2. Next, genes deferentially expressed between tumor grades, tumors with different hsa-miR-21-5p levels, and cell-line experiments were intersected with miRDB and TargetScan hsa-miR-21-5p targets.

### 4.6. Analysis of Publicly Available Single-Cell RNA-Sequencing Experiment

Publicly available scRNA-seq data GSE183655 [56] were analyzed for determining the expression of selected miRNA targets in cell types identified within the tumor tissue. Data were processed as described previously [11]. Count matrix was processed in Seurat (v5.1.0) [14], followed by quality control (feature count, total counts, and mitochondrial gene fraction). Two MSC-6 samples from the GSE183655 dataset (tumor and brain–tumor interface) were excluded upon quality checking. Doublets were identified with scds, and cells meeting the following thresholds were retained: ≥1000 counts, ≥500 features, mitochondrial fraction < 15%, and scds score < 1.5. Variable features were selected using the cv2 method [16], and datasets were integrated using Harmony v1.2.3. UMAP was used for dimensionality reduction, with informative principal components selected based on the Marchenko–Pastur distribution [17]. Cell types were assigned using previously established marker genes [11]. BisqueRNA [57] was utilized to estimate cell type population abundance in our bulk RNA-seq samples. Subsequently, CellChat [58] was utilized to establish cell–cell interactions in the tumor, brain–tumor interface, and dura. The weight of interactions and change in the weight of interactions were assessed. Furthermore, genes involved in the cell–cell signaling of a pathway were enriched in TargetScan hsa-miR-21-5p targets (fora in fgsea [55]). Cell types and genes involved in this signaling were identified and visualized.

### 4.7. In Vitro Cell Line Culture and Transfection with miRNA Mimics

The KT21-MG1 cells were a kind gift from Prof. Christian Mawrin and Prof. Elmar Kirches, and standard authentication for this cell line is not available. The cells were cultured in DMEM medium supplemented with 10% FBS and 1% Pen Strep (15140122, Gibco, Grand Island, NY, USA). MiScript miRNA mimics including hsa-miR-21-5p mimic (YM00470856-ADB, Qiagen), hsa-miR-21-3p mimic (YM00473093-ADB, Qiagen) and the negative control MiScript mimic (YM00479902-AGB; Qiagen) were used. 1.6 × 10^3^ cells were seeded per well of a 96-well plate in culture medium and transfected with 20 nM miRNA with 1% (*v*/*v*) HiPerFect Transfection Reagent (Qiagen), according to the manufacturer’s instructions. The next day, the culture medium was changed to fresh basic medium. The cells were harvested or subjected to in vitro tests at 48 h after transfection. All experiments were performed with mycoplasma-free cells in two biological replicates.

### 4.8. Functional In Vitro Assays

Cell viability was measured with MTT reagent (Sigma-Aldrich, St. Louis, MO, USA). Ten microliters of 5 mg/mL MTT stock solution were added to wells and cells were incubated for 4 h at 37 °C in a cell culture incubator. Next, the medium was carefully removed and the cells were flooded with 100 μL DMSO, mixed and incubated at 37 °C for 15 min [5]. Absorbance was measured immediately at 540 nm on a microplate reader. Cell proliferation was measured with a BrdU cell proliferation ELISA kit (Sigma-Aldrich), as previously [5]. The cells were incubated with BrdU reagent for 6 h at 37 °C in a cell culture incubator. Absorbance was measured at 450 nm and 690 nm using a Victor 3 microplate reader (Perkin Elmer, Wellesley, MA, USA).

### 4.9. Luciferase Reporter Gene Assay

The 3′UTR fragments of the genes that interact with hsa-miR-21-5p were identified with TargetScan 8.0 [52]. Approximately forty nucleotide long 3′UTR fragments containing the putative interaction sites of *TIMP3*, *PIK3R1*, *BTG2*, *RHOB*, *SASH1*, *IL6R*, *IL6ST* and *TGFBR2* were cloned into pmirGLO Dual-Luciferase miRNA Target Expression Vector (Promega, Madison, WI, USA), according to the protocol recommended by the plasmid vector supplier. Oligonucleotide sequences of 3′UTR inserts are listed in Supplementary Table S1. KT21-MG1 cells (1.6 × 10^3^ /well) were seeded onto a 96-well plate in 100 µL of medium. The next day, the cells were transfected with 100 ng of plasmid vector (3′UTR-containing or a control empty vector), using 0.25% (*v*/*v*) lipofectamine 3000 (Invitrogen) in 10 µL of DMEM. The cells were subsequently transfected with either hsa-miR-21-5p or hsa-miR-21-3p mimic (YM00470856-ADB and YM00473093-ADB, respectively; Qiagen) or the negative control mimic (YM00479902-ADB, Qiagen) in a final concentration of 20 nM using HiPerFect Reagent (Qiagen). Luciferase activity was measured with a Dual-Glo Luciferase Assay System (Promega) 48 h after transfection.

### 4.10. Immunohistochemistry

Immunohistochemistry was performed on 4 μm FFPE tissue sections using an EnVision detection system (DAKO, Glostrup, Denmark), as already described [5]. Briefly, tissue sections were deparaffinized in xylene, rehydrated through graded ethanol, and subjected to heat-induced epitope retrieval in a target retrieval solution (pH 6, DAKO) at 96 °C for 30 min. Endogenous peroxidase activity was blocked with a blocker of endogenous peroxidase (DAKO), and sections were incubated with mouse monoclonal anti–IL-6Rα (clone H-7, Santa Cruz, Dallas, TX, USA; 1:50) or anti-CD163 (clone MRQ-26, Cell Marque, Rocklin, CA, USA; ready-to-use) for 1 h at room temperature. Immunoreactivity was visualized using 3,3′-diaminobenzidine (DAB; DAKO) and nuclei were counterstained with hematoxylin.

### 4.11. Statistical Analysis

Quantitative continuous variables were tested for normal distribution using the Shapiro–Wilk test. When comparing two groups, a two-sided unpaired t-test was used for comparing the data with a normal distribution, while a two-sided Mann–Whitney U-test was used otherwise. When comparing more than two groups, one-way ANOVA (for variables with a normal distribution) or the Kruskal–Wallis test was used, followed by post hoc analysis with Benjamini–Hochberg correction. Spearman correlation was used for correlation analysis. Significance threshold α = 0.05 was adopted. R environment (v4.2.2) and GraphPad Prism 6.07 (GraphPad Software) were used for analysis and data visualization.

## Figures and Tables

**Table 1:** Characteristics of the patients.

Clinical Feature	
Number of patients	62
Females	40/62 (64.5%)
Males	22/62 (35.5%)
Age at surgery (years; median (range))	63 (38–90)
Meningioma subtype	
WHO grade I	32/62 (51.6%)
Meningothelial	19/62 (30.6.9%)
Fibrous	13/62 (21%)
WHO grade II, Atypical	18/62 (29%)
WHO grade III, Anaplastic	12/62 (19.35%)

## Data Availability

The HM450K DNA methylation data are available on the Gene Expression Omnibus (GEO) (accession GSE241956); the RNA sequencing datasets generated for this study are available from GEO under accession number GSE309391. The publicly available scRNA-seq dataset GSE183655 was used.

## Supplementary Materials

The following supporting information can be downloaded at: https://www.mdpi.com/article/10.3390/ijms27104403/s1.

## Abbreviations

The following abbreviations are used in this manuscript:

BAM	Border-associated macrophage
BTI	Brain-tumor interface
FFPE	Formalin-fixed paraffin-embedded
miRNA	MicroRNA
RNA-seq	RNA sequencing
scRNA-seq	Single-cell RNA sequencing
WHO	World Health Organization
UTR	Untranslated region

## References

[1] Low J.T., Ostrom Q.T., Cioffi G., Neff C., Waite K.A., Kruchko C., Barnholtz-Sloan J.S. (2022). Primary Brain and Other Central Nervous System Tumors in the United States (2014–2018): A Summary of the CBTRUS Statistical Report for Clinicians. Neurooncol. Pract..

[2] Louis D.N., Sahm F., Perry A., von Deimling A., Claus E.B., Mawrin C., Brastianos P.K., Meningiomas S.S., Brat D.J., Ellison D.W., Figarella-Branger D., Hawkins C., Louis D.N., Ng H.K., Perry A., Pfister S.M., Refeinberger G., Soffietti R. (2021). Central Nervous System Tumours. WHO Classification of Tumours.

[3] Robert S.M., Vetsa S., Nadar A., Vasandani S., Youngblood M.W., Gorelick E., Jin L., Marianayagam N., Erson-Omay E.Z., Günel M. (2022). The Integrated Multiomic Diagnosis of Sporadic Meningiomas: A Review of Its Clinical Implications. J. Neurooncol..

[4] He S., Pham M.H., Pease M., Zada G., Giannotta S.L., Wang K., Mack W.J. (2013). A Review of Epigenetic and Gene Expression Alterations Associated with Intracranial Meningiomas. Neurosurg. Focus.

[5] Kober P., Mossakowska B.J., Rusetska N., Baluszek S., Grecka E., Konopiński R., Matyja E., Oziębło A., Mandat T., Bujko M. (2023). Epigenetic Downregulation of Hsa-MiR-193b-3p Increases Cyclin D1 Expression Level and Cell Proliferation in Human Meningiomas. Int. J. Mol. Sci..

[6] Peng Y., Croce C.M. (2016). The Role of MicroRNAs in Human Cancer. Signal Transduct. Target. Ther..

[7] Zhang B., Pan X., Cobb G.P., Anderson T.A. (2007). MicroRNAs as Oncogenes and Tumor Suppressors. Dev. Biol..

[8] Wang L., Chen S., Liu Y., Zhang H., Ren N., Ma R., He Z. (2020). The Biological and Diagnostic Roles of MicroRNAs in Meningiomas. Rev. Neurosci..

[9] Hashemi M., Mirdamadi M.S.A., Talebi Y., Khaniabad N., Banaei G., Daneii P., Gholami S., Ghorbani A., Tavakolpournegari A., Farsani Z.M. (2023). Pre-Clinical and Clinical Importance of MiR-21 in Human Cancers: Tumorigenesis, Therapy Response, Delivery Approaches and Targeting Agents. Pharmacol. Res..

[10] Lu J., Tan T., Zhu L., Dong H., Xian R. (2020). Hypomethylation Causes MIR21 Overexpression in Tumors. Mol. Ther. Oncolytics.

[11] Baluszek S., Kober P., Myśliwy I., Oziębło A., Mandat T., Jeżewski M.P., Bujko M. (2025). Determining the Biological Features of Aggressive Meningioma Growth with Transcriptomic Profiling. Cancers.

[12] Zhan L., Mu Z., Jiang H., Zhang S., Pang Y., Jin H., Chen J., Jia C., Guo H. (2023). MiR-21-5p Protects against Ischemic Stroke by Targeting IL-6R. Ann. Transl. Med..

[13] Borja-Gonzalez M., Casas-Martinez J.C., McDonagh B., Goljanek-Whysall K. (2020). Inflamma-MiR-21 Negatively Regulates Myogenesis during Ageing. Antioxidants.

[14] Wang W., Yuan X., Xu A., Zhu X., Zhan Y., Wang S., Liu M. (2018). Human Cancer Cells Suppress Behaviors of Endothelial Progenitor Cells through MiR-21 Targeting IL6R. Microvasc. Res..

[15] Glaich O., Parikh S., Bell R.E., Mekahel K., Donyo M., Leader Y., Shayevitch R., Sheinboim D., Yannai S., Hollander D. (2019). DNA Methylation Directs MicroRNA Biogenesis in Mammalian Cells. Nat. Commun..

[16] Morales S., Monzo M., Navarro A. (2017). Epigenetic Regulation Mechanisms of MicroRNA Expression. Biomol. Concepts.

[17] Saviana M., Le P., Micalo L., Del Valle-Morales D., Romano G., Acunzo M., Li H., Nana-Sinkam P. (2023). Crosstalk between MiRNAs and DNA Methylation in Cancer. Genes.

[18] Ortiz I.M.D.P., Barros-Filho M.C., Dos Reis M.B., Beltrami C.M., Marchi F.A., Kuasne H., do Canto L.M., de Mello J.B.H., Abildgaard C., Pinto C.A.L. (2018). Loss of DNA Methylation Is Related to Increased Expression of MiR-21 and MiR-146b in Papillary Thyroid Carcinoma. Clin. Epigenet..

[19] Boresowicz J., Kober P., Rusetska N., Maksymowicz M., Paziewska A., Dabrowska M., Zeber-Lubecka N., Kunicki J., Bonicki W., Ostrowski J. (2020). DNA Methylation Influences miRNA Expression in Gonadotroph Pituitary Tumors. Life.

[20] El-Gewely M., Andreassen M., Walquist M., Ursvik A., Knutsen E., Nystad M., Coucheron D., Myrmel K., Hennig R., Johansen S. (2016). Differentially Expressed MicroRNAs in Meningiomas Grades I and II Suggest Shared Biomarkers with Malignant Tumors. Cancers.

[21] Galani V., Alexiou G.A., Miliaras G., Dimitriadis E., Triantafyllou E., Galani A., Goussia A., Kanavaros P., Trangas T. (2015). Expression of Stem Cell Marker Nestin and MicroRNA-21 in Meningiomas. Turk. Neurosurg..

[22] Rhim J., Baek W., Seo Y., Kim J.H. (2022). From Molecular Mechanisms to Therapeutics: Understanding MicroRNA-21 in Cancer. Cells.

[23] Mishra S., Deng J.J., Gowda P.S., Rao M.K., Lin C.L., Chen C.L., Huang T., Sun L.Z. (2014). Androgen Receptor and MicroRNA-21 Axis Downregulates Transforming Growth Factor Beta Receptor II (TGFBR2) Expression in Prostate Cancer. Oncogene.

[24] Zhao Q., Chen S., Zhu Z., Yu L., Ren Y., Jiang M., Weng J., Li B. (2018). MiR-21 Promotes EGF-Induced Pancreatic Cancer Cell Proliferation by Targeting Spry2. Cell Death Dis..

[25] Farasati Far B., Vakili K., Fathi M., Yaghoobpoor S., Bhia M., Naimi- Jamal M.R. (2023). The Role of MicroRNA-21 (MiR-21) in Pathogenesis, Diagnosis, and Prognosis of Gastrointestinal Cancers: A Review. Life Sci..

[26] Wang A.Z., Bowman-Kirigin J.A., Desai R., Kang L.I., Patel P.R., Patel B., Khan S.M., Bender D., Marlin M.C., Liu J. (2022). Single-Cell Profiling of Human Dura and Meningioma Reveals Cellular Meningeal Landscape and Insights into Meningioma Immune Response. Genome Med..

[27] Sun R., Jiang H. (2024). Border-Associated Macrophages in the Central Nervous System. J. Neuroinflamm..

[28] Kierdorf K., Masuda T., Jordão M.J.C., Prinz M. (2019). Macrophages at CNS Interfaces: Ontogeny and Function in Health and Disease. Nat. Rev. Neurosci..

[29] Lin F., Yin H.B., Li X.Y., Zhu G.M., He W.Y., Gou X. (2020). Bladder Cancer Cell-Secreted Exosomal MiR-21 Activates the PI3K/AKT Pathway in Macrophages to Promote Cancer Progression. Int. J. Oncol..

[30] Jin J., Yu G. (2022). Hypoxic Lung Cancer Cell-Derived Exosomal MiR-21 Mediates Macrophage M2 Polarization and Promotes Cancer Cell Proliferation through Targeting IRF1. World J. Surg. Oncol..

[31] Yu H., Pan J., Zheng S., Cai D., Luo A., Xia Z., Huang J. (2023). Hepatocellular Carcinoma Cell-Derived Exosomal MiR-21-5p Induces Macrophage M2 Polarization by Targeting RhoB. Int. J. Mol. Sci..

[32] Tiong T.Y., Chan M.L., Wang C.H., Yadav V.K., Pikatan N.W., Fong I.H., Yeh C.T., Kuo K.T., Huang W.C. (2023). Exosomal MiR-21 Determines Lung-to-Brain Metastasis Specificity through the DGKB/ERK Axis within the Tumor Microenvironment. Life Sci..

[33] Hu Z., You L., Hu S., Yu L., Gao Y., Li L., Zhang S. (2024). Hepatocellular Carcinoma Cell-Derived Exosomal MiR-21-5p Promotes the Polarization of Tumor-Related Macrophages (TAMs) through SP1/XBP1 and Affects the Progression of Hepatocellular Carcinoma. Int. Immunopharmacol..

[34] Qiao L., Dong C., Jia W., Sun G. (2024). RAB5A in Triple-Negative Breast Cancer: A Critical Role in Macrophage Reshaping in an Exosomal MiR-21-Dependent Manner. Endocr. Relat. Cancer.

[35] Zeboudj L., Sideris-Lampretsas G., Silva R., Al-Mudaris S., Picco F., Fox S., Chambers D., Malcangio M. (2023). Silencing MiR-21-5p in Sensory Neurons Reverses Neuropathic Allodynia via Activation of TGF-β-Related Pathway in Macrophages. J. Clin. Investig..

[36] Zhang Z., Hu J., Ishihara M., Sharrow A.C., Flora K., He Y., Wu L. (2022). The MiRNA-21-5p Payload in Exosomes from M2 Macrophages Drives Tumor Cell Aggression via PTEN/Akt Signaling in Renal Cell Carcinoma. Int. J. Mol. Sci..

[37] Chang J., Li H., Zhu Z., Mei P., Hu W., Xiong X., Tao J. (2022). MicroRNA-21-5p from M2 Macrophage-Derived Extracellular Vesicles Promotes the Differentiation and Activity of Pancreatic Cancer Stem Cells by Mediating KLF3. Cell Biol. Toxicol..

[38] Park K.J., Kang S.H., Chae Y.S., Yu M.O., Cho T.H., Suh J.K., Lee H.K., Chung Y.G. (2010). Influence of Interleukin-6 on the Development of Peritumoral Brain Edema in Meningiomas: Laboratory Investigation. J. Neurosurg..

[39] Schrell U.M.H., Koch H.U., Marschalek R., Schrauzer T., Anders M., Adams E., Fahlbusch R. (1998). Formation of Autocrine Loops in Human Cerebral Meningioma Tissue by Leukemia Inhibitor Factor, Interleukin-6, and Oncostatin M: Inhibition of Meningioma Cell Growth in Vitro by Recombinant Oncostatin M. J. Neurosurg..

[40] Fan H., Song L., Fan J., Ma J., Li X., Zhang J., Hu J., Wu Z., Zhang D., Wang L. (2023). Decoding Meningioma Heterogeneity and Neoplastic Cell—Macrophage Interaction through Single-Cell Transcriptome Profiling across Pathological Grades. J. Transl. Med..

[41] Liu Y., Wang D., Li Z., Li X., Jin M., Jia N., Cui X., Hu G., Tang T., Yu Q. (2022). Pan-Cancer Analysis on the Role of PIK3R1 and PIK3R2 in Human Tumors. Sci. Rep..

[42] Jiang K., Liu P., Xu H., Liang D., Fang K., Du S., Cheng W., Ye L., Liu T., Zhang X. (2020). SASH1 Suppresses Triple-Negative Breast Cancer Cell Invasion through YAP-ARHGAP42-Actin Axis. Oncogene.

[43] Zaoui K., Duhamel S. (2023). RhoB as a Tumor Suppressor: It’s All about Localization. Eur. J. Cell Biol..

[44] Wanting Y., Peizheng Y., Yan L., Yinfeng Y., Jinghui W. (2023). Research Progress of BTG2 as a Tumor Prognostic Factor. Arch. Cancer Sci. Ther..

[45] Boye A. (2021). A Cytokine in Turmoil: Transforming Growth Factor Beta in Cancer. Biomed. Pharmacother..

[46] Barski D., Wolter M., Reifenberger G., Riemenschneider M.J. (2010). Hypermethylation and Transcriptional Downregulation of the TIMP3 Gene Is Associated with Allelic Loss on 22q12.3 and Malignancy in Meningiomas. Brain Pathol..

[47] Johnson M.D. (2017). Transforming Growth Factor Beta Family in the Pathogenesis of Meningiomas. World Neurosurg..

[48] Mao B., Xiao H., Zhang Z., Wang D., Wang G. (2015). MicroRNA-21 Regulates the Expression of BTG2 in HepG2 Liver Cancer Cells. Mol. Med. Rep..

[49] Yan Q., Liu J., Liu Y., Wen Z., Jin D., Wang F., Gao L. (2024). Tumor-Associated Macrophage-Derived Exosomal MiR21-5p Promotes Tumor Angiogenesis by Regulating YAP1/HIF-1α Axis in Head and Neck Squamous Cell Carcinoma. Cell. Mol. Life Sci..

[50] Bujko M., Kober P., Rusetska N., Wakuła M., Goryca K., Grecka E., Matyja E., Neska J., Mandat T., Bonicki W. (2016). Aberrant DNA Methylation of Alternative Promoter of DLC1 Isoform 1 in Meningiomas. J. Neurooncol..

[51] Fortin J.P., Triche T.J., Hansen K.D. (2017). Preprocessing, Normalization and Integration of the Illumina HumanMethylationEPIC Array with Minfi. Bioinformatics.

[52] McGeary S.E., Lin K.S., Shi C.Y., Pham T.M., Bisaria N., Kelley G.M., Bartel D.P. (2019). The biochemical basis of microRNA targeting efficacy. Science.

[53] Chen Y., Wang X. (2020). MiRDB: An Online Database for Prediction of Functional MicroRNA Targets. Nucleic Acids Res..

[54] Castanza A.S., Recla J.M., Eby D., Thorvaldsdóttir H., Bult C.J., Mesirov J.P. (2023). Extending Support for Mouse Data in the Molecular Signatures Database (MSigDB). Nat. Methods.

[55] Korotkevich G., Sukhov V., Budin N., Shpak B., Artyomov M.N., Sergushichev A. (2016). Fast Gene Set Enrichment Analysis. BioRxiv.

[56] Choudhury A., Magill S.T., Eaton C.D., Prager B.C., Chen W.C., Cady M.A., Seo K., Lucas C.H.G., Casey-Clyde T.J., Vasudevan H.N. (2022). Meningioma DNA Methylation Groups Identify Biological Drivers and Therapeutic Vulnerabilities. Nat. Genet..

[57] Jew B., Alvarez M., Rahmani E., Miao Z., Ko A., Garske K.M., Sul J.H., Pietiläinen K.H., Pajukanta P., Halperin E. (2020). Accurate Estimation of Cell Composition in Bulk Expression through Robust Integration of Single-Cell Information. Nat. Commun..

[58] Jin S., Guerrero-Juarez C.F., Zhang L., Chang I., Ramos R., Kuan C.H., Myung P., Plikus M.V., Nie Q. (2021). Inference and Analysis of Cell-Cell Communication Using CellChat. Nat. Commun..

