# Supplementary material for: DNA Hypomethylation of MIR21 Drives Hsa-miR-21-5p Expression in High-Grade Meningiomas and Reshapes Transcriptomic Signatures of Oncogenic Pathways and Intercellular Communication
Source: Int J Mol Sci. 2026 May 15;27(10):4403. doi: 10.3390/ijms27104403 (PMC13206978; doi:10.3390/ijms27104403)
Supplement: Supplementary file 1 [file ijms-27-04403-s001.zip › Supplementary Figure S1.pdf]

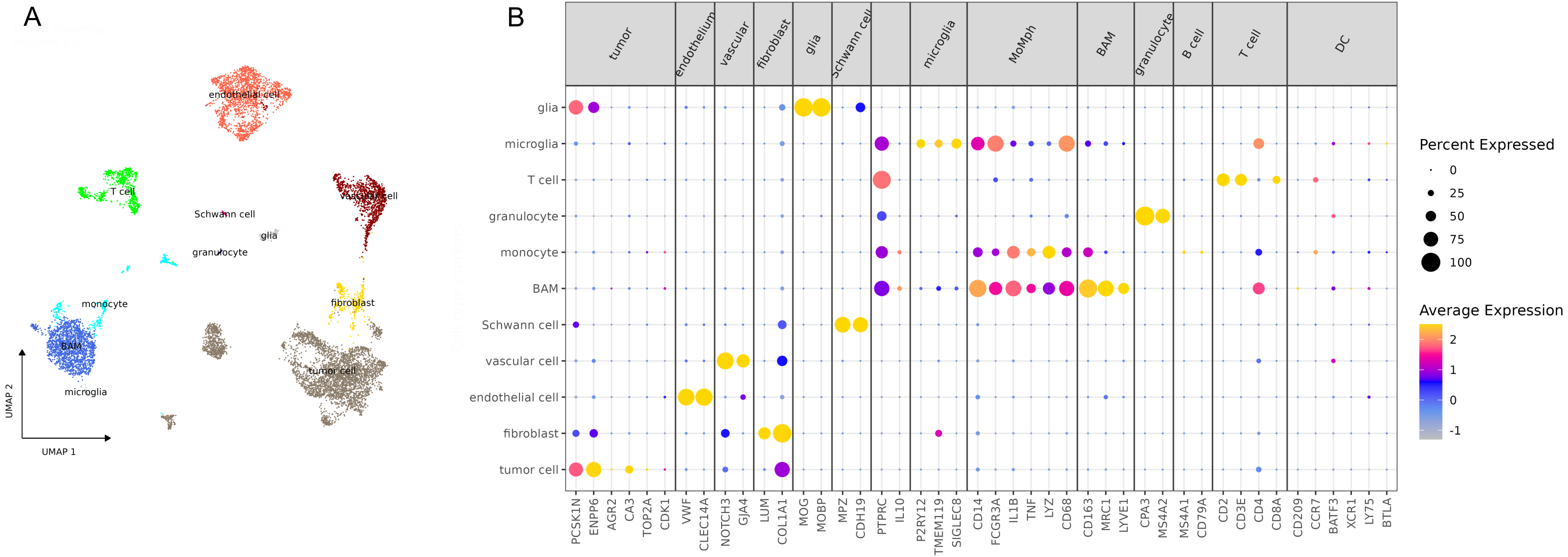

Supplementary Figure S1 Identification of the cellular subpopulation in meningioma tissue using deposited scRNAseq dataset (GSE183655). A) Clustering individual cells according to genes expression. B) Functional identification of each cellular cluster with a set of well-established marker gene.
